# Diversity and Antimicrobial Resistance Profiles of ESBL-Producing *Escherichia coli* in Surface Waters of Albania

**DOI:** 10.3390/pathogens15070737

**Published:** 2026-07-14

**Authors:** Florian Plaku, Ilir Kusi, Esmeralda Dushku, Anastasia Paraskeva, Virginia Giantzi, Erinda Lika, Fatbardh Sallaku, Theofilos Papadopoulos, Elena Tsavea, Charalampos Kotzamanidis

**Affiliations:** 1 Department of Clinical Subjects, Faculty of Veterinary Medicine, Agricultural University of Tirana (UBT), 1029 Tirana, Albania; fplaku@ubt.edu.al; 2 Department of Veterinary Preclinical Modules, Faculty of Veterinary Medicine, Agricultural University of Tirana (UBT), 1029 Tirana, Albania; ikusi@ubt.edu.al (I.K.); elika@ubt.edu.al (E.L.); 3 Hellenic Agricultural Organization-DIMITRA, Veterinary Research Institute of Thessaloniki, 57001 Thermi, Greece; nesmeral@bio.auth.gr (E.D.); anastasiaapar@gmail.com (A.P.); vgiantzi@elgo.gr (V.G.); thpapadopoulos@elgo.gr (T.P.); elenats89@hotmail.com (E.T.); 4 Department of Environment and Natural Resources (DENR), Faculty of Agriculture and Environment (FBM), Agricultural University of Tirana (UBT), 1029 Tirana, Albania; fsallaku@ubt.edu.al

**Keywords:** ESBL, antimicrobial resistance, β-lactamase genes, genotyping, surface water, phylogenetic groups

## Abstract

This study presents the first comprehensive molecular characterization of *Escherichia coli* producing extended-spectrum beta-lactamases (ESBL-Ec) in surface waters in Albania, focusing on the Shkumbini river. Antimicrobial resistance (AMR) in aquatic ecosystems poses a significant threat to public health, yet data from Albania remain scarce. Thirty water samples were collected from six locations near Elbasan between September 2022 and February 2024. Following the WHO Tricycle protocol, 52 ESBL-Ec isolates were recovered and characterized for antimicrobial susceptibility, biofilm formation, resistance genotypes and clonal relatedness via pulsed-field gel electrophoresis (PFGE). ESBL-Ec was detected in 80% of the samples analyzed, with 94.2% of the isolates classified as multidrug-resistant (MDR). High resistance frequencies were observed for ampicillin (98.1%) and cefotaxime (86.5%), while 7.7% of the isolates displayed colistin resistance associated with the *mcr*-3 gene. The *bla*_CTX-M-1_ genotype was the most prevalent (57.7%), and almost half of the isolates harbored multiple ESBL genes. Phylogroup A (46.2%) predominated, followed by the high-risk extraintestinal lineages B2 (23.1%) and D (11.5%). PFGE revealed high genetic heterogeneity, with 51 distinct pulsotypes indicating multiple sources of contamination, such as untreated municipal, agricultural and industrial waste. Additionally, 55.8% of the isolates were capable of forming biofilms. These results highlight the critical role of the Shkumbini river as a reservoir for highly resistant pathogens and emphasize the urgent need for integrated environmental surveillance and improved wastewater management in Albania.

## 1. Introduction

Antimicrobial resistance is a critical global public health threat of the 21st century, challenging effective treatment across human and veterinary medicine and increasing morbidity, mortality, and healthcare costs [1]. The rapid dissemination of antimicrobial resistance genes (ARGs) among bacteria is primarily driven by the remarkable genomic plasticity of these microorganisms. This plasticity facilitates the acquisition of ARGs through horizontal gene transfer (HGT), a process that is facilitated by mobile genetic elements such as plasmids, integrons, transposons and insertion sequences [2,3].

In the field of AMR dissemination, the emergence of plasmid-mediated extended-spectrum beta-lactamases (ESBLs) has become a matter of significant concern, threatening the efficacy of third-generation cephalosporins for the treatment of multidrug-resistant Gram-negative infections [4]. While *Escherichia coli* is a primary indicator for global AMR surveillance, it is crucial to recognize that other members of the Enterobacteriaceae family, such as *Klebsiella* spp. and *Enterobacter* spp., are also significant ESBL producers with major clinical and epidemiological impact [5].

Among Gram-negative pathogens, *E. coli* occupies a central position due to its dual role as a ubiquitous commensal organism and a major cause of both community- and hospital-acquired infections. E. coli has been identified as the indicator organism of choice in the context of environmental antimicrobial resistance (AMR) surveillance, as outlined in the WHO Tricycle protocol. This selection is primarily influenced by the ability of *E. coli* to serve as an indicator of fecal contamination and the subsequent transmission pathways it can reveal. The species exhibits extensive clonal diversity, and recent evidence highlights the potential of commensal strains to function as critical reservoirs for ARGs [6]. Extraintestinal pathogenic *E. coli* (ExPEC), including uropathogenic *E. coli* (UPEC), possess a range of virulence-associated factors, including adhesins, toxins, iron acquisition systems, protectins, and capsule-associated factors, which are usually encoded on pathogenicity islands (PAIs) and plasmids. These factors contribute to the bacteria’s ability to colonise, persist, and invade host tissues [7,8]. While ExPEC and uropathogenic *E. coli* UPEC are frequently linked to urinary tract infections, which have recently emerged as one of the most prevalent infectious diseases [9], virulence and resistance are not restricted to these groups. Commensal strains are known to frequently harbor clinically relevant genes and virulence determinants that are traditionally associated with distinct pathotypes, facilitating the silent spread of resistance within the environment and the microbiota of the host [10].

In recent decades, a substantial increase in the prevalence of *E. coli* strains that produce ESBLs has been observed in both clinical and non-clinical settings [11]. The global dissemination of ESBL-producing *E. coli* (ESBL-Ec) is predominantly driven by the propagation of CTX-M-type β-lactamases, plasmid-mediated enzymes that confer resistance to a broad spectrum of β-lactams and are readily transferable among bacterial hosts [12]. Furthermore, of particular concern is the recent rise of *E. coli* isolates that are resistant to colistin, often mediated by the plasmid-borne *mcr* genes, which facilitate the horizontal transfer of resistance to this antibiotic [13]. Since then, numerous *mcr* variants have been detected in a variety of settings, including clinical [14], agricultural, and environmental [15] contexts worldwide, while the co-occurrence of ESBL and *mcr* genes within the same isolates is particularly alarming, as it creates strains with severely limited therapeutic options and increases the likelihood of environmental-to-human transmission [16,17].

While ESBL-Ec was initially regarded primarily as a clinical problem, there has been a substantial shift in understanding, due to the recognition that resistant bacteria circulate across human, animal and environmental reservoirs. A plethora of studies have demonstrated the ubiquity of ESBL-Ec strains in livestock, wildlife, companion animals, food products, soil, and aquatic ecosystems [18,19]. It has been increasingly recognised that environmental compartments, particularly surface waters, function as pivotal interfaces for the accumulation, persistence, and dissemination of resistant bacteria and resistance genes [20]. A growing body of evidence shows that ESBL-Ec is prevalent in rivers worldwide, often carrying clinically relevant resistance determinants such as blaCTX-M-1, blaCTX-M-14, and blaCTX-M-15 [21,22]. The presence of human-associated sequence types, including ST131, ST10, ST38, and ST40, in surface waters underscores the contribution of wastewater contamination and highlights the potential for environmentally derived *E. coli* to pose risks to human and animal health. Surface water isolates frequently exhibit multidrug resistance, including co-resistance to fluoroquinolones, aminoglycosides, tetracyclines, and sulphonamides, illustrating the complexity of aquatic AMR ecology. Furthermore, the presence of plasmids, integrons, and transposons in aquatic *E. coli* has been demonstrated to promote horizontal gene transfer, thereby accelerating the dissemination of resistance determinants across bacterial populations [23,24,25]. In this context, the characterization of environmental ESBL-producing *E. coli* extends beyond the mere documentation of resistance frequencies. Simultaneous investigation of antimicrobial resistance profiles, ESBL genotypes, phylogenetic background, genetic diversity, and ecological traits such as biofilm formation provides insight into whether environmental isolates exhibit characteristics commonly associated with clinically relevant lineages.

In view of the role of rivers in the environmental dissemination of AMR, it is imperative that continuous surveillance and molecular characterization of indicator bacteria in these systems be implemented. In the context of the Western Balkans, the absence of established national surveillance programmes for antibiotic consumption and antimicrobial resistance in Albania severely limits informed clinical and public health responses [26,27]. Operating within a One Health framework, it was hypothesized that the Shkumbini River functions as a reservoir of genetically diverse multidrug-resistant ESBL-producing *E. coli*, including isolates belonging to phylogenetic groups frequently associated with extraintestinal pathogenic lineages. The objectives of this study were to (i) determine the prevalence of ESBL-producing *E. coli* in the Shkumbini river, (ii) characterize their antimicrobial susceptibility patterns and ESBL resistance genes, (iii) investigate their phylogenetic distribution, genetic diversity and biofilm-forming ability, and (iv) evaluate these findings collectively within a One Health perspective to establish baseline data for environmental antimicrobial resistance surveillance in Albania.

## 2. Materials and Methods

### 2.1. Description of the Study Location and Sample Collection

**Figure 1:**
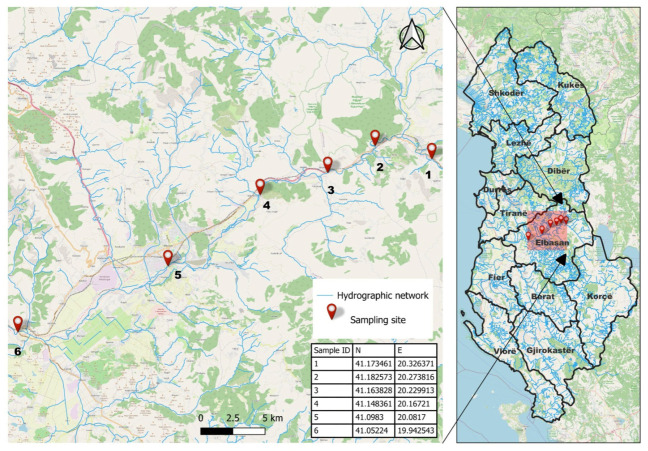
Location and map of the Shkumbin river. The sampling sites are indicated by their numbers along the Shkumbin river.

From September 2022 to February 2024, a total of 30 samples were collected at four-month intervals from six sampling locations along a section of the Shkumbini river near Elbasan. The Shkumbini river, a major waterway in Albania, is 181 km long with an average annual water flow of 61 cubic meters per second. It passes through urban centers, agricultural zones, and industrial areas before reaching the Adriatic Sea (Figure 1). Sampling points were selected both upstream and downstream of Elbasan, a city with a population of 296,000, based on the World Health Organization Tricycle Protocol for global surveillance on ESBL-Ec, which suggests methodology for site selection at the city level [28]. In all cases, 500 mL of water samples were collected in sterile plastic containers at a depth of approximately 30 cm below the water surface. The samples were then transported to the laboratory on ice and processed within 24 h of collection.

### 2.2. Collection of Isolates

The isolation of ESBL-Ec was performed using the membrane filtration method, following the World Health Organization Tricycle Protocol, a standardized approach for detecting ESBL-Ec [28]. Water samples of 100 mL were filtered through 0.45 μm filters, after which the filter membranes were placed on tryptone bile X-glucuronide agar (TBX) (Oxoid, Basingstoke, UK) plates supplemented with 4 µg/mL cefotaxime (CTX; Sigma-Aldrich, St. Louis, MI, USA). The plates were then incubated for 24–48 h at 37 °C. Colonies exhibiting blue or blue-green pigmentation on chromogenic agar were identified as potential ESBL-Ec and subjected to further investigation. In order to capture potential intra-sample diversity and avoid the selection of clonal duplicates, three morphologically diverse colonies from each plate were initially transferred to TBX agar and further processed to confirm species identification. This confirmation process involved assessment of indole production, using the BBL Dry SlideTM (BD) method, to detect the presence of the enzyme tryptophanase, which catalyzes the breakdown of tryptophan into indole, together with the determination of phenotypic evidence of ESBL production through the double disk synergy test (DDST) performed on Mueller-Hinton agar (Merck, Darmstadt, Germany), in accordance with EUCAST guidelines [29]. ESBL-Ec isolates were preserved at −70 °C in tryptic soy broth (TSB) (Oxoid, Basingstoke, UK), with the addition of 20% (*v*/*v*) glycerol, until further analysis. *E. coli* ATCC 25922 was used as the control strain for the methodology.

### 2.3. Antimicrobial Susceptibility Profiles of ESBL-Ec

A total of 15 antimicrobial agents were tested against ESBL-Ec isolates using the standard disc diffusion method on Mueller–Hinton agar (Merck, Darmstadt, Germany) with commercially available discs (Oxoid, Basingstoke, UK), except for colistin, which was tested by broth microdilution to determine the minimum inhibitory concentration (MIC).

The antimicrobial agents employed in this study represented multiple antibiotic classes. These included penicillins (ampicillin, AMP, 10 μg), penicillins/β-lactamase inhibitor combinations (amoxicillin–clavulanic acid, AMC, 30 μg), cephamycins (cefoxitin, FOX, 30 μg), cephalosporins (cefotaxime, CTX, 5 μg; ceftazidime, CAZ, 10 μg), monobactams (aztreonam, ATM, 30 μg), carbapenems (meropenem, MEM, 10 μg; imipenem, IMP, 10 μg), tetracyclines (tetracycline, TET, 30 μg), fluoroquinolones (ciprofloxacin, CIP, 5 μg), aminoglycosides (gentamicin, CN, 10 μg; streptomycin, S, 10 μg), phenicols (chloramphenicol, C, 30 μg), folate pathway inhibitors (sulfamethoxazole–trimethoprim, SXT, 25 μg), and polymyxins (colistin, COL).

The estimation of the antimicrobial susceptibility of the isolates was performed according to EUCAST breakpoint criteria [30], except for tetracycline, which was evaluated according to CLSI guidelines [31]. *E. coli* ATCC 25922 was included in each run as the quality control strain to validate disc potency and media quality according to standard EUCAST protocols [30]. Strains exhibiting resistance to three or more antibiotic classes were classified as MDR [32].

### 2.4. Assessment of Biofilm Formation Ability of ESBL-Ec

The biofilm-forming ability of ESBL-Ec isolates was evaluated using the semi-quantitative microtiter plate assay, as previously described [33], with minor modifications. Briefly, each isolate was cultivated overnight in TSB for 24–48 h at 37 °C, then diluted to 10^8^ CFU/mL (adjusted to a turbidity equivalent to a 0.5 McFarland standard) using TSB supplemented with 1% (*w*/*v*) glucose to enhance biofilm formation. A total of 200 µL of the diluted suspension was dispensed into triplicate wells of a sterile 96-well flat-bottom polystyrene microplate. Wells containing sterile broth alone served as negative controls. Subsequently, the plates were incubated at 37 °C for 24 h under static conditions with the objective of enabling the formation of biofilms. After incubation, the planktonic cells were gently removed, and the wells were washed three times with sterile phosphate-buffered saline (PBS, pH 7.2) to remove non-adherent bacteria. The remaining attached biofilms were stained by adding 100 μL of 0.3% (*w*/*v*) crystal violet solution for 15 min. Excess stain was removed by washing the plates under running distilled water, and the plates were air-dried at room temperature. The bound crystal violet was solubilized by adding 200 µL of 33% (*v*/*v*) glacial acetic acid to each well, after which the optical density (OD) of the adherent biofilms was measured at 570 nm using a microplate reader (Tecan Infinite M200 Pro, Grodig, Austria). Biofilm production was classified according to the optical density cut-off (ODc), defined as three standard deviations above the mean OD of the negative control. Isolates were categorized as non-biofilm producers (OD ≤ ODc), weak (ODc < OD ≤ 2 × ODc), moderate (2 × ODc < OD ≤ 4 × ODc), or strong biofilm producers (OD > 4 × ODc) [34]. Each assay was performed in triplicate, and mean OD values were calculated.

### 2.5. Pulsed-Field Gel Electrophoresis Genotyping

The clonal relatedness of ESBL-Ec isolates was assessed using pulsed-field gel electrophoresis (PFGE) following the standardized PulseNet protocol for *E. coli*, with *Xba*I (New England Biolabs, Beverly, MA, USA) as the restriction enzyme [35]. Restricted DNA fragments were separated using a CHEF-DR III instrument (Bio-Rad Laboratories Inc., Hercules, CA, USA). *Salmonella enterica* serovar Braenderup H9812 digested with *Xba*I was used as the molecular size standard, while PFGE patterns were digitally analyzed using the FPQuest (version 5, Bio-Rad Laboratories Pty Ltd., Hercules, CA, USA) software package.

### 2.6. Detection of Resistance Genes and Phylogenetic Typing

ESBL-Ec strains were characterized with respect to their phylogenetic group and the genetic basis of ESBL production. For this purpose, genomic DNA was extracted from overnight cultures using a commercial bacterial DNA extraction kit (Invitrogen, Carlsbad, CA, USA), according to the manufacturer’s instructions, and stored at −20 °C until use. 

Phylogenetic grouping of ESBL-Ec isolates was performed using a multiplex PCR method as previously described [36]. The isolates were assigned to groups A, B1, B2, C, D, E, or F, targeting the *arp*A, *chu*A, *yja*A, and TspE4.C2 genetic markers. The presence of major β-lactamase gene families associated with ESBL production in ESBL-Ec isolates was investigated by multiplex PCR. Detection of seven β-lactamase gene families, including *bla*_CTX-M-1_ group, *bla*_CTX-M-2_ group, *bla*_CTX-M-9_ group, *bla*_CTX-M_ group 8/25, *bla*_OXA-1-like_, *bla*_SHV_, and *bla*_TEM_, was achieved using specific primers and conditions as previously described [37]. Furthermore, ESBL-Ec isolates exhibiting resistance to colistin (R > 2 mg/L) were subjected to multiplex PCR analysis to ascertain the presence of *mcr*-1, -2, -3, -4, and -5 [38].

### 2.7. Statistical Analysis

The statistical analysis was conducted utilising XL-STAT (v.2023.1.5.1409, Addinsoft, New York, NY, USA). The frequency distribution of the biofilm-formation ability of ESBL-Ec was determined by means of contingency tables, according to phylogroup type. The relationships between categorical variables were subjected to an assessment using the chi-square test. It was considered that a *p*-value of less than 0.05 would indicate statistical significance. The analysis of PFGE patterns was conducted utilizing the BioNumerics software package (version 6.6; Applied Maths, Sint-Martins-Latem, Belgium). The PFGE profiles were subjected to comparison using the Dice correlation coefficient, with a maximum position tolerance of 1.5% and an optimization of 1.5%. A similarity clustering analysis was performed using the unweighted pair group method with arithmetic means (UPGMA), and a dendrogram was consequently generated.

## 3. Results

### 3.1. Prevalence of ESBL-Ec

A total of 52 isolates exhibiting ESBL production were recovered from water samples from the Shkumbini river. ESBL-Ec was found in 80.0% (24/30) of water samples originating from all sampling points.

### 3.2. Phenotypic Characterization of ESBL-Ec-Antibiotic Resistance Profiling and Biofilm Formation Ability

According to antimicrobial susceptibility testing of the 52 ESBL-Ec isolates, all displayed resistance to at least three of the tested antimicrobials, resulting in 42 distinct antimicrobial resistance profiles (ARPs). Among these ARPs, only one type (AMC CTX CAZ ATM CIP S SXT C AMP TET) was observed in five out of the six sampling points (Table 1).

Higher resistance frequencies were observed against penicillins (AMP, 98.1%), aminoglycosides (S, 98.1%; CN, 28.8%), cephalosporins (CTX, 86.5%; CAZ, 55.8%) and the monobactam aztreonam (ATM, 80.8%), followed by penicillin and β-lactamase inhibitors (AMC, 63.5%), tetracyclines (TET, 51.9%), phenicols (C, 48.1%), fluoroquinolones (CIP, 42.3%), folate pathway inhibitors (SXT, 36.5%), cephamycins (FOX, 11.5%), and carbapenems (MEM, 9.6%; IMP, 1.9%). Additionally, resistance to colistin (COL) was noted in four isolates (Table 1 and Table 2). Out of the 52 confirmed ESBL-Ec isolates, it was found that a striking 94.2% (49/52) were resistant to three or more different antimicrobial classes and could therefore be characterized as MDR, with a uniform distribution observed across nearly all sites. It was evident that sampling points 1, 2, and 3 exhibited frequencies of 100% MDR among the recovered isolates (Table 2).

The biofilm formation assay differentiated ESBL-Ec isolates into three categories: moderate, weak and non-producers (Table 2). The majority of isolates were characterized as weak biofilm producers (48.1%, 25/52), followed by isolates with no biofilm formation capacity (44.2%, 23/52) and moderate biofilm formation capacity (7.7%, 4/52) (Table 2). Chi-square test revealed that biofilm formation capacity of ESBL-Ec was not significantly associated with their phylogroups (chi-square =13.050; *p* = 0.367).

### 3.3. Distribution of ESBL Genotypes and Phylogenetic Groups

Among the 52 ESBL-Ec isolates, five of the seven gene families analyzed by conventional PCR were detected in 75% (39 out of 52) of the isolates with 43.6% (17 out of 39) of these harboring more than one gene. The *bla*_CTX-M-1_ gene family (57.7%; *n* = 30) was the most prevalent, followed by *bla*_TEM_ (30.8%; *n* = 16), *bla*_OXA-1-like_ (11.5%; *n* = 6) and *bla*_CTX-M-9_ (11.5%; *n* = 6), while the *bla*_CTX-M-2_, *bla*_CTX-M_ group 8/25, and *bla*_SHV_ genes were not detected. Regarding the presence of colistin resistance genes, the four isolates exhibiting the colistin-resistant phenotype were found to carry the *mcr*-3 gene. Analysis of the isolates’ phylogenetic grouping revealed that the majority were assigned to the Clermont phylogroup A (46.2%; 24/52), followed by B2 (23.1%; 12/52), D (11.5%; 6/52), B1 (9.6%; 5/52), C (5.8%; 3/52), and E (3.8%; 2/52). (Table 2).

### 3.4. ESBL-Ec Strains Genetic Relatedness

**Figure 2:**
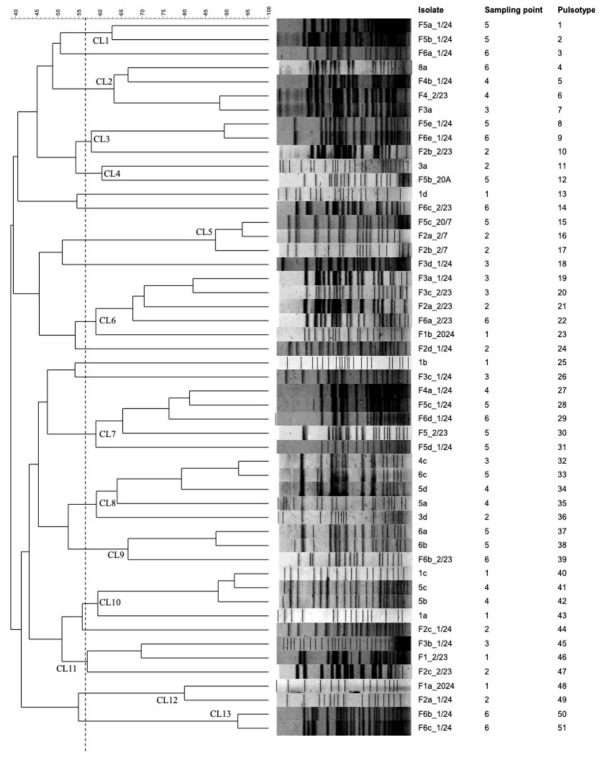
Dendrogram of *Xba*I PFGE macrorestriction patterns of ESBL-Ec, isolated from the Shkumbini river. The dendrogram is based on analysis by the unweighted pair group with the arithmetic averages clustering method. The clusters CL1-CL13 defined at a similarity level of 57% (dotted line).

Of the 52 ESBL-Ec isolates, 51 were successfully typed by PFGE following digestion with the restriction enzyme *Xba*I. This analysis yielded 51 distinct pulsotypes (Figure 2). At a similarity level of 57% or higher, most isolates (84.3%, 43/51) were grouped into 13 clusters (CL1-13), comprising isolates from different sampling points. Notably, no common pulsotypes were identified among isolates from the same or different sampling points.

## 4. Discussion

This study presents the first comprehensive phenotypic and molecular characterization of ESBL-Ec in surface waters of Albania, focusing on the Shkumbini river. Detection of ESBL-Ec in 80% of the analyzed water samples indicates widespread contamination of this aquatic ecosystem with antimicrobial-resistant bacteria. The Shkumbini river is considered highly polluted, flowing through urban settlements, agricultural areas, and industrial zones, all of which are potential sources of fecal contamination and antibiotic residues that may contribute to the persistence and proliferation of resistant bacteria in the aquatic environment [39,40]. The high prevalence of ESBL-Ec may also reflect the high daily doses per 1000 inhabitants of antibiotics consumed in Albania, due to high rates of self-purchasing of antibiotics in the country [41]. These findings provide further evidence supporting the hypothesis that surface waters serve as reservoirs and dissemination pathways for antimicrobial resistance determinants and resistant bacterial populations. The high prevalence of ESBL-Ec observed in this study is consistent with reports from river systems worldwide, where surface waters frequently receive untreated or partially treated municipal wastewater, hospital effluents, and agricultural runoff containing resistant microorganisms. Similarly high detection rates have been reported in river systems worldwide, suggesting that anthropogenic activities play a pivotal role in the environmental dissemination of ESBL-producing *Enterobacterales* [22,42].

By assessing the antimicrobial susceptibility profile of ESBL-Ec, a wide variation in resistance profiles was observed (42 ARPs among 52 isolates) within the Shkumbini river. This likely reflects differential selection pressures due to intensive use of antimicrobials; an increase in the combined consumption of cephalosporins and quinolones (22–37%) between 2011 and 2015 in Albania has been reported [35]. Moreover, the majority of isolates (94.2%) were classified as MDR, indicating resistance to three or more antimicrobial classes. Such high MDR rates have also been reported in ESBL-Ec recovered from rivers and wastewater treatment plant effluents in several regions of Europe and Asia, emphasizing the role of aquatic environments as reservoirs of clinically relevant resistance phenotypes [43].

Nearly all isolates demonstrated resistance to ampicillin, and a significant proportion showed resistance to third-generation cephalosporins, reflecting the selective pressure exerted by β-lactam antibiotic use. The high prevalence of resistance to ampicillin and third-generation cephalosporins among *E. coli* isolates from the Shkumbini river is a matter of profound clinical and epidemiological concern. Ampicillin is widely used to treat various infections in both animals and humans [44], while the WHO has classified third-generation cephalosporin-resistant *Enterobacterales* as “Critical Priority” pathogens due to their significant global health implications, including high rates of treatment failure and increased healthcare costs [45]. Furthermore, it has been demonstrated in previous studies that mobile genetic elements, including insertion sequences and plasmids, have the capacity to promote the co-selection and persistence of antimicrobial resistance determinants in conjunction with ExPEC-associated virulence genes. In particular, associations between ampicillin resistance, the genes coding for Afa/Dr adhesins, and insertion sequence-mediated genetic rearrangements have been described in UPEC [46,47,48,49], suggesting that acquisition of resistance may occur concurrently with virulence-associated traits that enhance bacterial persistence, invasion, and survival within host tissues [50].

The detection of carbapenem-resistant ESBL-Ec in the Shkumbini river, particularly resistance to meropenem (9.6%) and imipenem (1.9%), is a critical public health finding of this study, as it is associated with increased morbidity and mortality in clinical settings. Carbapenems are considered last-resort antibiotics for treating severe infections caused by multidrug-resistant Gram-negative bacteria [51]. The emergence of carbapenem resistance among ESBL-producing strains indicates the accumulation of multiple resistance mechanisms, often mediated by mobile genetic elements such as plasmids carrying carbapenemase genes, and significantly limits therapeutic options [52]. Although carbapenems are primarily reserved for human medicine and are not widely used in veterinary practice, previous studies have reported the isolation of *Enterobacterales* that are both ESBL-producing and carbapenem-resistant from surface waters, suggesting indirect selective pressures, likely driven by the discharge of hospital effluents, municipal wastewater, and inadequately treated sewage [53].

Of particular concern is the detection of colistin resistance in 7.7% (*n* = 4) of the ESBL-Ec isolates from the Shkumbini river, specifically associated with the carriage of the plasmid-mediated *mcr*-3 gene. Several studies have reported the emergence of mobile colistin resistance genes in environmental bacteria, which represents a significant public health concern because these determinants can be readily transferred among bacterial populations via horizontal gene transfer [54]. The co-occurrence of ESBL production and plasmid-mediated colistin resistance further reduces therapeutic options and increases the risk of environmental reservoirs contributing to the dissemination of highly resistant pathogens. In the context of the Shkumbini river, which receives untreated municipal wastewater from urban centres such as Elbasan and provides irrigation to agricultural land [39,55,56] the detection of multiple-resistant strains may reflect that, in Albania, *Enterobacteriaceae* isolated from farm animals show extremely high levels of resistance to multiple antimicrobials [57]. The Institute for Health Metrics and Evaluation (IHME) has reported that the number of AMR deaths in Albania exceeds the number of deaths from chronic respiratory diseases, digestive diseases, diabetes and kidney diseases, respiratory infections and tuberculosis [58]. Additionally, the main resistance challenge in the country appears to be linked with Gram-negative organisms, particularly ESBL-producing *Enterobacteriaceae* [59]. This underscores the significance of river waters as a prospective reservoir and environmental-to-human transmission route of these critical resistance determinants.

The molecular characterization of ESBL genes in this study revealed a clear predominance of the blaCTX-M-1 group, detected in more than half of the isolates (57.7%). CTX-M-type enzymes are currently the most widespread ESBLs globally and are frequently associated with both clinical and environmental *E. coli* strains. The predominance observed in this study is consistent with the findings from numerous environmental surveys. These surveys demonstrate that CTX-M-type β-lactamases, particularly those in the CTX-M-1 group, are the primary drivers of ESBL dissemination in aquatic ecosystems [60,61]. The detection of additional ESBL determinants such as *bla*_TEM_, *bla*_OXA-1-like_, and *bla*_CTX-M-9_ is consistent with previous studies showing the genetic diversity of β-lactamase genes circulating in this environment [2,62]. Genetic diversity analysis by PFGE revealed a high level of heterogeneity among the ESBL-Ec isolates; all strains exhibited distinct pulsotypes, and no identical PFGE profiles were observed among isolates collected from different sampling points, indicating that the river is exposed to a wide range of genetically distinct ESBL-Ec strains. Taken together, the results of the molecular characterization of the isolates, regarding both the genetic heterogeneity among the ESBL-Ec isolates and the genetic diversity of β-lactamase genes detected, indicate the introduction into the river of resistant bacteria from multiple contamination sources, such as domestic sewage, livestock operations, and industrial and hospital discharges [63,64].

In the present study, phylogenetic grouping and biofilm formation were evaluated as complementary epidemiological characteristics rather than direct indicators of virulence [65]. Phylogenetic analysis revealed that most isolates belonged to phylogroup A, followed by B2 and D. Phylogroup A is typically associated with commensal strains that are widely distributed in environmental reservoirs, while groups B2 and D are commonly linked to extraintestinal pathogenic *E. coli* (ExPEC) [66,67]. The ability to form biofilms is an important trait of bacteria that enhances their survival in aquatic environments [68] and enables them to cause persistent urinary tract infections [69]; assays to quantify biofilm formation have been suggested as a possible method for screening pathogenic enteroaggregative *E. coli* (EAEC) strains [66]. In the present study, almost half of the isolates exhibited weak biofilm-forming ability (48.1%), while a smaller proportion showed moderate biofilm production (7.7%). It is important to note that the production of biofilms alone cannot be used to infer pathogenic potential, because virulent and non-virulent E. coli strains may display similar biofilm phenotypes [70,71]. In the present study, the biofilm formation capacity of ESBL-Ec was not significantly associated with their phylogroups. Consequently, the ability of environmental ESBL-producing *E. coli* to form biofilms may contribute to their long-term persistence within aquatic ecosystems and increase opportunities for dissemination of antimicrobial resistance determinants [72,73]. It is noteworthy that sampling point 5 was identified as a pivotal location for the convergence of high-risk traits, exhibiting a high MDR frequency (90.9%), a substantial proportion of virulent B2/D lineages (63.7%), moderate and weak biofilm phenotypes, and the concentrated occurrence of the plasmid-mediated colistin resistance gene *mcr*-3. The structural overlay indicates an explicit correlation between point-source waste pressures in the vicinity of Elbasan and elevated public health risks.

## 5. Conclusions

The results of this study demonstrate that the Shkumbini river represents an important environmental reservoir of MDR and ESBL-Ec. The combination of high prevalence, diverse resistance gene profiles, and genetic heterogeneity suggests ongoing contamination from multiple anthropogenic sources. From a public health perspective, the presence of ESBL-producing, carbapenem- and colistin-resistant *E. coli* in surface waters raises concerns about potential human exposure through recreational water activities, crop irrigation, and contamination of drinking water resources. These findings highlight the importance of implementing integrated environmental surveillance programmes to monitor antimicrobial resistance in aquatic ecosystems. Despite providing valuable baseline data, this study is limited by the relatively small number of isolates analyzed, which precluded the assessment of potential correlations between antimicrobial resistance patterns and spatial or temporal factors such as sampling sites and seasonal variation. Further large-scale and longitudinal investigations are required to more accurately characterize the dynamics of antimicrobial resistance in aquatic environments. In countries such as Albania, where national AMR surveillance systems are still developing, environmental monitoring can provide valuable insights into the circulation and spread of resistant bacteria. It is recommended that future research initiatives encompass a more extensive surveillance programme, incorporating a greater diversity of *Enterobacteriaceae*, across a wider range of river systems, wastewater treatment facilities, and coastal environments. This approach will facilitate a more comprehensive understanding of the dynamics underlying the dissemination of antimicrobial resistance in the region. The pathogenic potential of the recovered isolates cannot be directly inferred because established ExPEC virulence determinants, including adhesins (e.g., *fim*H, *pap*, *afa*/*dra*), toxins (e.g., *hly*A, *cnf*1), iron acquisition systems (e.g., *iut*A, *fyu*A), and capsule-associated genes (e.g., *kps*MT II), were not investigated. Future studies integrating antimicrobial resistance profiling with molecular characterization of these virulence-associated genes, or whole-genome sequencing approaches, will be necessary to more accurately assess the potential human health significance of environmental ESBL-producing *E. coli* circulating in Albanian surface waters.

## Figures and Tables

**Table 1:** Phenotypic and genotypic characteristics of the ESBL-Ec isolated from the Shkumbini river.

Sampling Point	Period of Sampling	Isolates	Antimicrobial Resistance Profile ^a^															Biofilm ^b^	Phylogenetic Group ^c^	Antimicrobial Resistance Genes (PCR) ^d^							
			AMC	FOX	CTX	CAZ	ATM	CIP	S	CN	SXT	C	AMP	MEM	TET	IMP	COL			OXA Family	SHV Family	TEM Family	CTX-M Group 1	CTX-M Group 2	CTX-M Group 9	CTX-M Group 8/25	*mcr*-1,-2,-3,-4,-5
1	September 2022	1a	S	S	S	S	S	S	**R**	**R**	S	S	**R**	**R**	S	**R**	S	M	A								
		1b	S	S	S	S	S	**R**	S	**R**	S	S	**R**	S	S	S	S	W	A								
		1c	S	S	S	S	S	S	**R**	**R**	S	S	**R**	S	S	S	S	W	C								
		1d	S	S	S	S	S	S	**R**	**R**	S	S	**R**	S	S	S	S	W	A								
2		3a	S	S	S	S	**R**	S	**R**	**R**	S	S	**R**	S	S	S	S	W	A								
		3d	R	S	R	**R**	S	S	**R**	S	S	S	**R**	S	S	S	S	NB	A								
3		4c	R	S	R	S	**R**	R	**R**	S	S	**R**	**R**	S	**R**	S	S	W	C								
4		5a	R	S	R	S	**R**	R	**R**	S	**R**	**R**	**R**	S	**R**	S	S	W	C								
		5b	S	S	S	**R**	S	S	**R**	S	S	S	**R**	S	**R**	S	S	NB	A								
		5c	S	S	S	S	S	S	**R**	S	S	S	**R**	S	S	S	S	NB	A								
		5d	S	S	R	S	**R**	**R**	**R**	**R**	S	**R**	**R**	S	**R**	S	S	NB	A								
5		6a	S	S	R	S	S	S	**R**	**R**	S	s	**R**	S	S	S	S	NB	A								
		6b	S	S	R	**R**	**R**	S	**R**	S	S	S	**R**	**R**	S	S	S	W	A								
		6c	S	S	R	S	S	**R**	**R**	S	S	**R**	**R**	S	**R**	S	S	W	A								
6		8a	S	S	R	S	**R**	S	**R**	**R**	S	S	S	S	**R**	S	S	NB	B2								
1	February 2023	F1_2/23	S	S	R	**R**	**R**	S	**R**	S	S	**R**	**R**	S	**R**	S	**R**	W	D								
2		F2a_2/23	**R**	S	R	**R**	**R**	**R**	**R**	S	S	**R**	**R**	S	S	S	S	NB	B2								
		F2b_2/23	**R**	S	R	**R**	**R**	S	**R**	**R**	**R**	**R**	**R**	S	**R**	S	S	NB	B2								
		F2c_2/23	S	S	R	**R**	**R**	S	**R**	S	**R**	**R**	**R**	S	**R**	S	S	NB	A								
3		F3c_2/23	**R**	S	R	**R**	**R**	**R**	**R**	S	**R**	**R**	**R**	S	**R**	S	S	W	B2								
5		F5_2/23	**R**	S	R	**R**	**R**	**R**	**R**	S	**R**	**R**	**R**	S	**R**	S	S	NB	A								
6		F6a_2/23	**R**	S	R	**R**	**R**	**R**	**R**	S	**R**	**R**	**R**	S	S	S	S	W	B2								
		F6b_2/23	**R**	S	R	**R**	**R**	**R**	**R**	S	**R**	**R**	**R**	S	**R**	S	S	W	A								
		F6c_2/23	**R**	S	R	**R**	**R**	**R**	**R**	S	**R**	**R**	**R**	S	**R**	S	S	NB	A								
4		F4_2/23	**R**	S	R	**R**	**R**	**R**	**R**	S	**R**	**R**	**R**	S	**R**	S	S	W	B2								
5		F5b_20A	**R**	**R**	R	**R**	**R**	**R**	**R**	**R**	**R**	**R**	**R**	**R**	**R**	S	**R**	W	A								
2	June 2023	F2a_2/7	**R**	S	R	**R**	**R**	**R**	**R**	S	**R**	**R**	**R**	S	**R**	S	S	W	D								
		F2b_2/7	**R**	**R**	R	**R**	**R**	**R**	**R**	S	S	**R**	**R**	**R**	**R**	S	**R**	NB	D								
3		F3a	**R**	S	R	**R**	**R**	**R**	**R**	S	**R**	S	**R**	S	**R**	S	S	W	B2								
5		F5c_20/7	**R**	**R**	R	S	**R**	**R**	**R**	S	**R**	**R**	**R**	**R**	**R**	S	**R**	M	D								
1	February 2024	F1a_2024	**R**	S	R	S	**R**	**R**	**R**	**R**	S	S	**R**	S	**R**	S	S	W	A								
		F1b_2024	**R**	S	R	**R**	**R**	**R**	**R**	S	S	S	**R**	S	S	S	S	NB	B2								
2		F2a_1/24	**R**	S	R	**R**	**R**	S	**R**	S	**R**	**R**	**R**	S	**R**	S	S	NB	A								
		F2b_1/24	**R**	S	R	S	**R**	S	**R**	S	S	S	**R**	S	S	S	S	NB	B1								
		F2c_1/24	S	S	R	S	**R**	S	**R**	S	**R**	S	**R**	S	S	S	S	W	D								
		F2d_1/24	**R**	S	R	**R**	**R**	**R**	**R**	S	**R**	S	**R**	S	**R**	S	S	W	E								
3		F3a_1/24	**R**	S	R	S	**R**	S	**R**	S	S	S	**R**	S	S	S	S	W	B1								
		F3b_1/24	S	S	R	**R**	**R**	S	**R**	S	**R**	S	**R**	S	**R**	S	S	W	A								
		F3c_1/24	**R**	**R**	R	**R**	**R**	S	**R**	**R**	**R**	S	**R**	S	**R**	S	S	W	A								
		F3d_1/24	**R**	**R**	R	**R**	**R**	S	**R**	**R**	S	**R**	**R**	S	**R**	S	S	NB	E								
4		F4a_1/24	**R**	S	R	**R**	**R**	S	**R**	S	S	S	**R**	S	S	S	S	M	A								
		F4b_1/24	**R**	S	R	**R**	**R**	S	**R**	**R**	S	S	**R**	S	S	S	S	W	B2								
5		F5a_1/24	**R**	**R**	R	**R**	**R**	**R**	**R**	**R**	**R**	S	**R**	S	S	S	S	NB	B2								
		F5b_1/24	S	S	R	S	S	**R**	**R**	S	S	S	**R**	S	S	S	S	W	B2								
		F5c_1/24	S	S	R	S	**R**	S	**R**	S	S	S	**R**	S	S	S	S	NB	A								
		F5d_1/24	**R**	S	R	S	**R**	S	**R**	S	S	**R**	**R**	S	S	S	S	W	B2								
		F5e_1/24	S	S	R	**R**	**R**	S	**R**	S	S	S	**R**	S	**R**	S	S	NB	A								
6		F6a_1/24	**R**	S	R	S	**R**	S	**R**	S	S	**R**	**R**	S	S	S	S	NB	B1								
		F6b_1/24	**R**	S	R	**R**	**R**	S	**R**	S	S	**R**	**R**	S	**R**	S	S	NB	B1								
		F6c_1/24	**R**	S	R	S	**R**	S	**R**	S	S	S	**R**	S	S	S	S	M	B1								
		F6d_1/24	**R**	S	R	**R**	**R**	S	**R**	S	S	**R**	**R**	S	S	S	S	NB	A								
		F6e_1/24	**R**	S	R	S	**R**	S	**R**	S	S	**R**	**R**	S	S	S	S	NB	D								

^a^ Red square, resistant to a specific antimicrobial; white square sensitive to a specific antimicrobial; Antimicrobial acronyms: AMC, amoxicillin/clavulanic acid; FOX, cefoxitin; CTX, cefotaxime; CAZ, ceftazidime; ATM, aztreonam; CIP, ciprofloxacin; S, streptomycin; CN, gentamicin; SXT, sulfamethoxazole/trimethoprim; C, chloramphenicol; AMP, ampicillin; MEM, meropenem; TET, tetracycline; IMP, imipenem; COL, colistin. ^b^ Ability to form biofilms according to Borges [32]: W, weak biofilm producer; NB, no biofilm producer; M, moderate biofilm producer. ^c^ Phylogenetic group determined by PCR assay [34]. ^d^ Green square, positive for a specific gene; white square, negative for a specific gene.

**Table 2:** Integrated overview of ESBL-*E. coli* distribution, phylogroups, multidrug resistance (MDR), and biofilm traits along the Shkumbini River.

Sampling Point	No. of Isolates (% of Total Isolates)	Dominant Phylogroups (% of Site Isolates) ^a^	MDR Isolates *n* (%)	Dominant Resistance Traits/Classes ^b^	Dominant ESBL/*mcr* Genotypes Detected	Biofilm Phenotype Distribution ^c^
1	7 (13.5%)	A (57.1%), B2 (14.3%), C (14.3%)	7 (100%)	Penicillins (AMP), Aminoglycosides (S, CN), Cephalosporins (CTX, CAZ), Carbapenems (MEM/IMP), Polymyxins (COL)	*bla* _CTX-M-1_ group, *bla*_TEM_, *mcr*-3 (1 isolate)	W: 4 (57.1%) M: 0 (0%) NB: 3 (42.9%)
2	11 (21.2%)	A (45.5%), B2 (27.3%), D (27.3%)	11 (100%)	Penicillins (AMP), Cephalosporins (CTX, CAZ), Monobactams (ATM), Phenicols (C), Polymyxins (COL), Carbapenems (MEM)	*bla*_CTX-M-1_ group, *bla*_TEM_, *bla*_OXA-1_-_like_, *mcr*-3 (1 isolate)	W: 4 (36.4%) M: 0 (0%) NB: 7 (63.6%)
3	7 (13.5%)	A (42.9%), B1 (28.6%), E (28.6%)	7 (100%)	Penicillins (AMP), Cephalosporins (CTX, CAZ), Tetracyclines (TET), Folate Inhibitors (SXT)	*bla*_CTX-M-1_ group, *bla*_TEM_, *bla*_CTX-M-9_ group	W: 5 (71.4%) M: 0 (0%) NB: 2 (28.6%)
4	7 (13.5%)	A (57.1%), B2 (42.9%)	6 (85.7%)	Penicillins (AMP), Aminoglycosides (S), Cephalosporins (CTX, CAZ), Monobactams (ATM)	*bla*_CTX-M-1_ group, *bla*_TEM_,	W: 3 (42.9%) M: 1 (14.3%) NB: 3 (42.9%)
5	11 (21.2%)	B2 (45.5%), A (36.4%), D (18.2%)	10 (90.9%)	Penicillins (AMP), Cephalosporins (CTX), Aminoglycosides (S), Fluoroquinolones (CIP), Carbapenems (MEM), Polymyxins (COL)	*bla*_CTX-M-1_ group, *bla*_TEM_, *bla*_OXA-1-like_, *mcr*-3 (2 isolates)	W: 6 (54.5%) M: 1 (9.1%) NB: 4 (36.4%)
6	9 (17.3%)	B1 (44.4%), A (33.3%), D (22.2%)	8 (88.9%)	Penicillins (AMP), Cephalosporins (CTX), Monobactams (ATM), Phenicols (C)	*bla*_CTX-M-1_ group, *bla* _CTX-M-9_ group	W: 3 (33.3%) M: 2 (22.2%) NB: 4 (44.4%)
Total	52	A: 24 (46.2%), B2: 12 (23.1%), D: 6 (11.5%), B1: 5 (9.6%), C: 3 (5.8%), E: 2 (3.8%)	49 (94.2%)	AMP/S (98.1%), CTX (86.5%), ATM (80.8%), AMC (63.5%)	*bla*_CTX-M-1_ (57.7%), *bla*_TEM_ (30.8%), *bla*_OXA-1-like_ (11.5%), *bla*_CTX-M-9_ (11.5%)	W: 25 (48.1%) M: 4 (7.7%) NB: 23 (44.2%)

^a^ Phylogenetic group determined by PCR assay [36]. ^b^ Antimicrobial acronyms: AMC, amoxicillin/clavulanic acid; FOX, cefoxitin; CTX, cefotaxime; CAZ, ceftazidime; ATM, aztreonam; CIP, ciprofloxacin; S, streptomycin; CN, gentamicin; SXT, sulfamethoxazole/trimethoprim; C, chloramphenicol; AMP, ampicillin; MEM, meropenem; TET, tetracycline; IMP, imipenem; COL, colistin. ^c^ Ability to form biofilms according to Borges [34]: W, weak biofilm producer; NB, no biofilm producer; M, moderate biofilm producer.

## Data Availability

The original contributions presented in this study are included in the article. Further inquiries can be directed to the corresponding author.
